# New species and records of Pseudochironomini Sæther, 1977 (Diptera, Chironomidae) from the Dominican Republic

**DOI:** 10.3897/BDJ.11.e111925

**Published:** 2023-10-24

**Authors:** Trond Andersen, Amelie Höcherl, Jeremy Hübner, Caroline Chimeno, Xiaolong Lin, Viktor A. Baranov

**Affiliations:** 1 University of Bergen, Bergen, Norway University of Bergen Bergen Norway; 2 Bavarian State Collection of Zoology, Munich, Germany Bavarian State Collection of Zoology Munich Germany; 3 Shanghai Ocean University, Shanghai, China Shanghai Ocean University Shanghai China; 4 Estación Biológica de Doñana-CSIC, Sevilla, Spain Estación Biológica de Doñana-CSIC Sevilla Spain

**Keywords:** Chironominae, Pseudochironomini, *Pseudochironomus*, *Manoa*, new species, new records, Dominican Republic, Neotropical Region

## Abstract

**Background:**

Pseudochironomini is a relatively small and poorly-studied tribe of subfamily Chironominae (Diptera, Chironomidae).

**New information:**

*Pseudochironomusruthae* Andersen & Baranov **sp. nov.** is described and figured, based on a single male collected in a light trap at Matadero, Dominican Republic. The species can be separated from its congeners by the combination of the following characters: wing without dark bands, dorsocentrals in partly double row and apex of superior volsella rounded. The species is the first *Pseudochironomus* species to be formally recorded and described from the Caribbean. In addition, a new record of *Manoapahayokeensis* Jacobsen & Perry, 2002 from the Dominican Republic is given. One specimen was DNA-barcoded and the barcode is given.

## Introduction

The tribe Pseudochironomini within the subfamily Chironominae was established by Sæther (1977a). The tribe is characterised by having a black comb on fore tibia, similar to the combs on mid- and hind tibiae and, in the male, the median volsella is generally present. Originally, the genera *Aedokritus* Roback, 1958, *Manoa* Fittkau, 1963, *Megacentron* Freeman, 1961, *Pseudochironomus* Malloch, 1915, *Psilochironomus* Sublette, 1966 and *Riethia* Kieffer, 1917 were included in the tribe (Sæther 1977a). However, *Psilochironomus* is now considered to be a *nomen dubium* (*Spies and Reiss 1996*). Later, Andersen (2016) added the genus *Madachironomus* Andersen, 2016, based on two species from Madagascar to the tribe. According to Cranston (2003), the tribe Pseudochironomini might not be monophyletic.

The genus *Pseudochironomus* was described by Malloch (1915), based on *P.richardsoni* Malloch, 1915 from Illinois, USA. *Pseudochironomus* is the most species-rich genus in the tribe. About 11 species are known from the Nearctic Region and one or two species from the Palaearctic Region (Epler et al. 2013). The Nearctic species were reviewed by Sæther (1977b). From the Neotropical Region, Kieffer (1925) described *P. viridis Kieffer 1925* from Argentina. Roback (1960) recorded the species from Peru and re-described the male. Later, Paggi and Rodriguez-Garay (2015) re-described and figured the male, female, pupa and larva in more detail, based on material from Argentina. Recently, Shimabukuro et al. (2017) and Trivinho-Strixino and Shimabukuro (2018) described eight new *Pseudochironomus* species from Brazil and Andersen (2023) described a new species from Mexico and Costa Rica. Additionally, Watson and Heyn (1993) reported at least 11 (likely undescribed) species of *Pseudochironomus* as well as Pseudochironomuscf.viridis.

The genus *Manoa* was described by Fittkau (1963), based on *M.obscura* Fittkau, 1963 from Brazil. Later, *M.tangae* Andersen & Sæther, 1997 (Andersen and Sæther 1997) from Tanzania, *M.pahayokeensis* Jacobsen & Perry, 2002 (Jacobsen and Perry 2002) from Florida, USA and *M.xianjuensis* Qi & Lin, 2017 (Qi et al. 2017) from China were added.

No *Pseudochironomus* species have been described from the Caribbean so far, but Silva et al. (2015) listed the genus from the Dominican Republic, as well as reporting *Manoapahayokeensis* Jacobsen & Perry, 2002. Below, we describe a new species of *Pseudochironomus* from the country. It can be separated from other *Pseudochironomus* species by having the wing without dark bands, dorsocentrals in a partly double row and the apex of superior volsella is rounded. We also report a new record of *M.pahayokeensis* from the Dominican Republic.

## Materials and methods

When collected, the specimens were preserved in 80% ethanol and later slide-mounted in Euparal following the procedure outlined by Sæther (1969). The morphological nomenclature follows Sæther (1980).

The specimens were collected under the collection permit of Ministro de Medio Ambiente y Recursos Naturales of Dominican Republic for the project “Long peace of the Caribbean – have biota of the Dominican Republic really remained virtually unchanged for over 13 million years?” and were exported under export permit # VAPB-07404. The holotype of *Pseudochironomusruthae* Andersen & Baranov sp. nov. is deposited in the collection at the Department of Natural History, University Museum of Bergen, Norway [ZMBN]. The material of *Manoapahayokeensis* Jacobsen & Perry is housed in the Bavarian State Collection of Zoology (SNSB-ZSM).

The DNA was extracted from the material at the SNSB molecular lab using the NucleoSpin 96 Tissue (Macherey-Nagel) DNA extraction kit after having undergone an overnight lysis at 56°C. The COI barcodes were amplified using the LepF1 and LepR1 standard barcoding primers (Leray et al. 2013) using a Biometra Thermocycler (Analytik Jena) and the following PCR conditions: 2 min at 94°C; first cycle set (5 repeats): 30 s denaturation at 94°C, 40 s annealing at 45°C and 60 s extension at 72°C. Second cycle set (35 repeats): 30 s denaturation at 94°C, 40 s annealing at 51°C and 60 s extension at 72°C; final elongation 10 min at 72°C. The PCR products were cleaned-up using the ExoSAP-IT Express (Thermo Fisher) Kit, then sent to the LMU Sequencing Service at Biozentrum (Martinsried, Germany) for Sanger sequencing. Every specimen’s COI barcode was sequenced as a forward and reverse strand. The traces were edited in BioEdit Hall (1999) and a consensus sequence of the forward and reverse strands was obtained and uploaded to Barcode of Life Data Systems (www.boldsystems.org) (Ratnasingham and Hebert 2007). The original traces were uploaded as well.

## Taxon treatments

### Pseudochironomus ruthae

Andersen & Baranov sp. nov.

F0322AB7-A93A-5EB2-A425-76F13A5D3094

A4FFA263-932D-4734-A24E-44764E3E0AFC

#### Materials

- **Type status:** Holotype. **Occurrence:** occurrenceID: A5E2B29A-388F-5FCA-9FF3-728AD814291D; **Taxon:** scientificName: *Pseudochironomusruthae* Andersen & Baranov; **Location:** continent: Central America; waterBody: Caribbean; islandGroup: Greater Antilles; island: Hispaniola; country: Dominican Republic; countryCode: DO; stateProvince: Azua; municipality: Guayabal; locality: El Naranjito Matadero; minimumElevationInMeters: 1400; maximumElevationInMeters: 1400; verbatimLatitude: 18 40 27.70N; verbatimLongitude: 70 42 03.30W; georeferencedBy: Amelie Höcherl; **Event:** samplingProtocol: light trap, Amelie Höcherl; eventDate: 10 November 2019; startDayOfYear: 10 Nov; endDayOfYear: 10 Nov; year: 2019; month: November; day: 10; habitat: Location next to a nearly dry stream in a forested area; **Record Level:** type: PhysicalObject; language: en; rights: Reconocimiento 3.0 España (CC BY 3.0 ES); rightsHolder: CSIC; institutionID: University Museum of Bergen, Norway; institutionCode: ZMBN; basisOfRecord: PreservedSpecimen

#### Description

**Figure 1.**
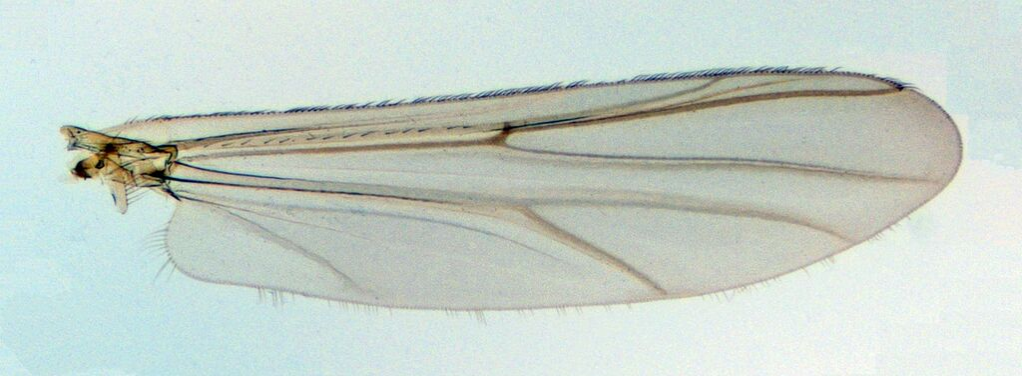
*Pseudochironomusruthae* Andersen & Baranov **sp. nov.**, male. Wing.

**Male** (n = 1). Total length 4.64 mm. Wing length 2.12 mm (Fig. 1). Total length/wing length 2.19. Wing length/length of profemur 2.24.

Colouration: Head and thorax dark brown; abdomen and legs brown, abdominal tergites VII and VIII with posterior lighter brown subrectangular field.

Antenna: AR 2.28. Ultimate flagellomere 1004 μm long.

Head: Temporals apparently about 25 in double to multiple rows. Clypeus with about 20 setae. Tentorium and stipes not measurable. Palp segment lengths (in μm): 67, 94, 162, 201 and 267. Third palpomere with all together four sensilla clavata in two pits apically, longest 21 μm long.

Thorax: Antepronotum with seven setae. With about 37 dorsocentrals in mainly double rows. Pre-alars six in single line. Scutellum with about 28 setae.

Wing (Fig. 1): VR 1.06. Brachiolum with two setae, R with 15 setae, remaining veins and cells bare. Squama with 17 setae.

Legs: Spur of fore tibia 66 μm long, spurs of mid-tibia 79 μm and 68 μm long, spurs of hind tibia 90 μm and 75 μm long. Width at apex of fore tibia 62 μm, of mid-tibia 77 μm, of hind tibia 80 μm. Sensilla chaetica five on ta1 of both mid- and hind legs. Lengths and proportions of legs as in Table 1.

**Figure 2.**
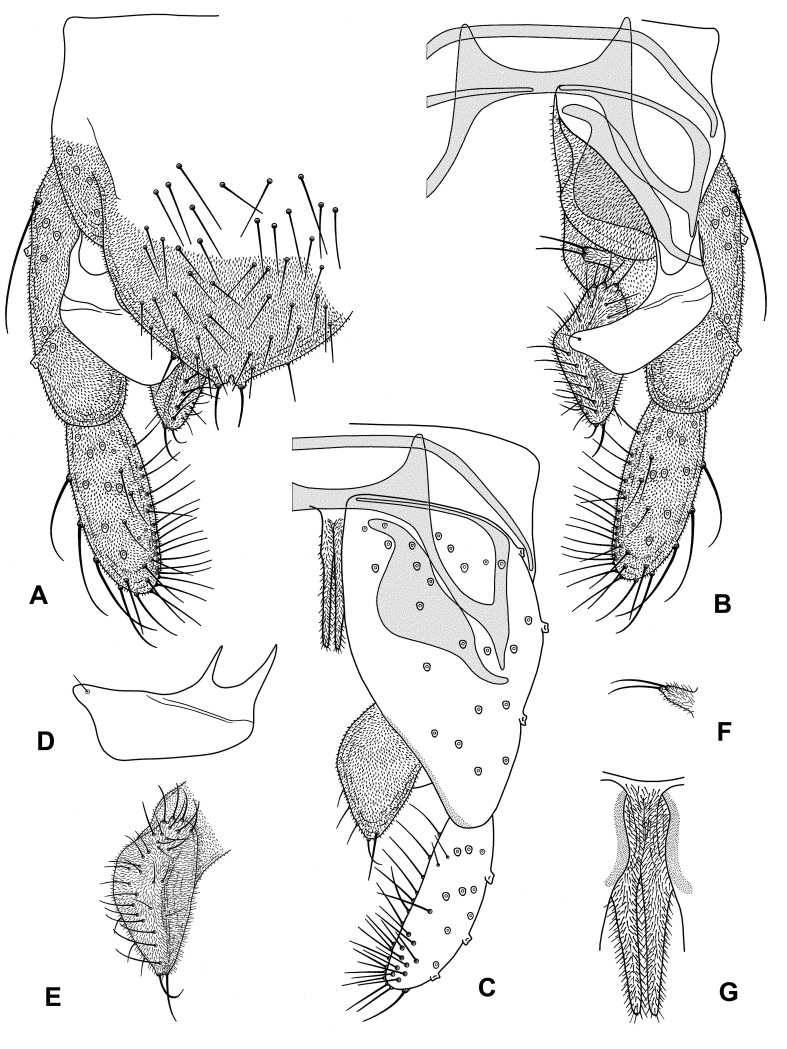
*Pseudochironomusruthae* Andersen & Baranov **sp. nov.**, male. **a** Hypopygium, dorsal view; **b** Hypopygium with tergite IX removed, dorsal view; **c** Hypopygium, ventral view; **d** Superior volsella; **e** Inferior volsella; **f** Median volsella; **g** Pars ventralis.

Hypopygium (Fig. 2): Tergite IX with broadly rounded posterior margin, with small median notch with single strong setae on weak tubercle to each side; with 43 additional dorsal and marginal setae. Laterosternite IX with four setae. Transverse sternapodeme straight, 138 μm long, with strong oral projections. Phallapodeme 164 μm long. Gonocoxite 248 μm long. Superior volsella pediform, 113 μm long, 41 μm wide medially, with rounded projection anteriomesally with single sensilla subapically. Inferior volsella with subovate apical part and base set of centrline of the hypopigium; apical part 104 μm wide, mesally with ridge with row of setae. Median volsella digitate, 17 μm long, with two strong and one weaker seta apically. Pars ventralis 88 μm long, narrow, apparently split at 1/3, apical parts 11 μm wide at base. Gonostylus 133 μm long. HR = 1.87; HV = 3.49.

**Female and immatures.** Unknown.

#### Diagnosis

The species can be separated from its congeners by the combination of the following characters: wing without dark bands, dorsocentrals in partly double row and apex of superior volsella rounded.

#### Etymology

Named after Ruth Bastardo who runs the aquatic ecology group and without whom the specimen would not have been collected.

#### Distribution

Dominican Republic: El Naranjito Matadero, 18°40'27.70"N, 70°42'03.30"E.

#### Taxon discussion

The new species is quite distinct from other described Neotropical *Pseudochironomus* species by the combination of having a wing without dark bands, dorsocentrals in a partly double row and apex of the superior volsella bluntly rounded. It is most similar to the Nearctic *Pseudochironomusrichardsoni* Malloch, 1915 (Sæther 1977a), but differs on the narrower median volsella of *Pseudochironomusruthae* (vs. wider conical one of the *P.richardsoni*), more triangular inferior of volsella of *P.ruthae*, rather than an elongated one of *P.richardsoni*; additionally, *P.ruthae* has a lower number of dorsocentrals (37 vs. 59 in *P.richardsoni)* (*Sæther 1977a*). In the key to the Pseudochironomini occurring in Brazil, it falls next to *P.jordensis* Shimabukuro & Trivinho-Strixino, 2017 and *P.mocidade* Shimabukuro & Trivinho-Strixino, 2017, as the wing of the adult male is longer than 3.0 mm and the posterior margin of terigte IX has a median notch (Trivinho-Strixino and Shimabukuro 2018, Shimabukuro et al. 2017). Hovewer, *P.ruthae* is distinct from all other South American *Pseudochironomus* species by possessing a superior volsella with a blunt, rounded apex, instead of the elongated beak-like protrusions, apparent in both *P.jordensis* and *P.mocidade*. Unfortunately, we were not able to DNA sequence the new species and its phylogenetic relationships to other representatives of the genus will require further elucidation.

### Manoa pahayokeensis

Jacobsen & Perry, 2002

DDA78357-E056-56F9-A923-788998033929

#### Materials

- **Type status:** Other material. **Occurrence:** recordedBy: Viktor Baranov; individualCount: 1; sex: male; lifeStage: adult; occurrenceID: AB3D8DCE-D9FB-54E6-BE0C-8665712880A1; **Taxon:** scientificName: Manoapahayokeensis; **Location:** country: Dominican Republic; stateProvince: Monseńor Nouel Province; locality: Blanco; verbatimElevation: 950 m; verbatimCoordinates: 18 88 49.18N 70 50 74.59W; decimalLatitude: 18.884918; decimalLongitude: -70.507459; georeferenceProtocol: GPS; **Identification:** identifiedBy: Viktor Baranov; dateIdentified: 2021; **Event:** samplingProtocol: sweeping; eventDate: 30th of November 2019; **Record Level:** language: en; institutionID: Bavarian State Collection of Zoology; institutionCode: SNSB-ZSM; collectionCode: Diptera; basisOfRecord: PreservedSpecimen
- **Type status:** Other material. **Occurrence:** recordedBy: Viktor Baranov; individualCount: 1; sex: male; lifeStage: adult; occurrenceID: 82D39763-390C-50F7-A17F-DB00C4D6D050; **Taxon:** scientificName: Manoapahayokeensis; **Location:** country: Dominican Republic; stateProvince: Monseńor Nouel Province; locality: Blanco; verbatimElevation: 950 m; verbatimCoordinates: 18 88 49.18N 70 50 74.59W; decimalLatitude: 18.884918; decimalLongitude: -70.507459; georeferenceProtocol: GPS; **Identification:** identifiedBy: Viktor Baranov; dateIdentified: 2021; **Event:** samplingProtocol: sweeping; eventDate: 30th of November 2019; **Record Level:** language: en; institutionID: Bavarian State Collection of Zoology; institutionCode: SNSB-ZSM; collectionCode: Diptera; basisOfRecord: PreservedSpecimen
- **Type status:** Other material. **Occurrence:** recordedBy: Viktor Baranov; individualCount: 1; sex: female; lifeStage: adult; occurrenceID: D342B44B-3635-5B5B-B8DB-265AA98B7F6D; **Taxon:** scientificName: Manoapahayokeensis; **Location:** country: Dominican Republic; stateProvince: Monseńor Nouel Province; locality: Blanco; verbatimElevation: 950 m; verbatimCoordinates: 18 88 49.18N 70 50 74.59W; decimalLatitude: 18.884918; decimalLongitude: -70.507459; georeferenceProtocol: GPS; **Identification:** identifiedBy: Viktor Baranov; dateIdentified: 2021; **Event:** samplingProtocol: sweeping; eventDate: 30th of November 2019; **Record Level:** language: en; institutionID: Bavarian State Collection of Zoology; institutionCode: SNSB-ZSM; collectionCode: Diptera; basisOfRecord: PreservedSpecimen
- **Type status:** Other material. **Occurrence:** recordedBy: Viktor Baranov; individualCount: 1; sex: female; lifeStage: adult; occurrenceID: 774717AE-D8EE-59E8-9651-36419A680D8B; **Taxon:** scientificName: Manoapahayokeensis; **Location:** country: Dominican Republic; stateProvince: Monseńor Nouel Province; locality: Blanco; verbatimElevation: 950 m; verbatimCoordinates: 18 88 49.18N 70 50 74.59W; decimalLatitude: 18.884918; decimalLongitude: -70.507459; georeferenceProtocol: GPS; **Identification:** identifiedBy: Viktor Baranov; dateIdentified: 2021; **Event:** samplingProtocol: sweeping; eventDate: 30th of November 2019; **Record Level:** language: en; institutionID: Bavarian State Collection of Zoology; institutionCode: SNSB-ZSM; collectionCode: Diptera; basisOfRecord: PreservedSpecimen

#### Taxon discussion

**Figure 3.**
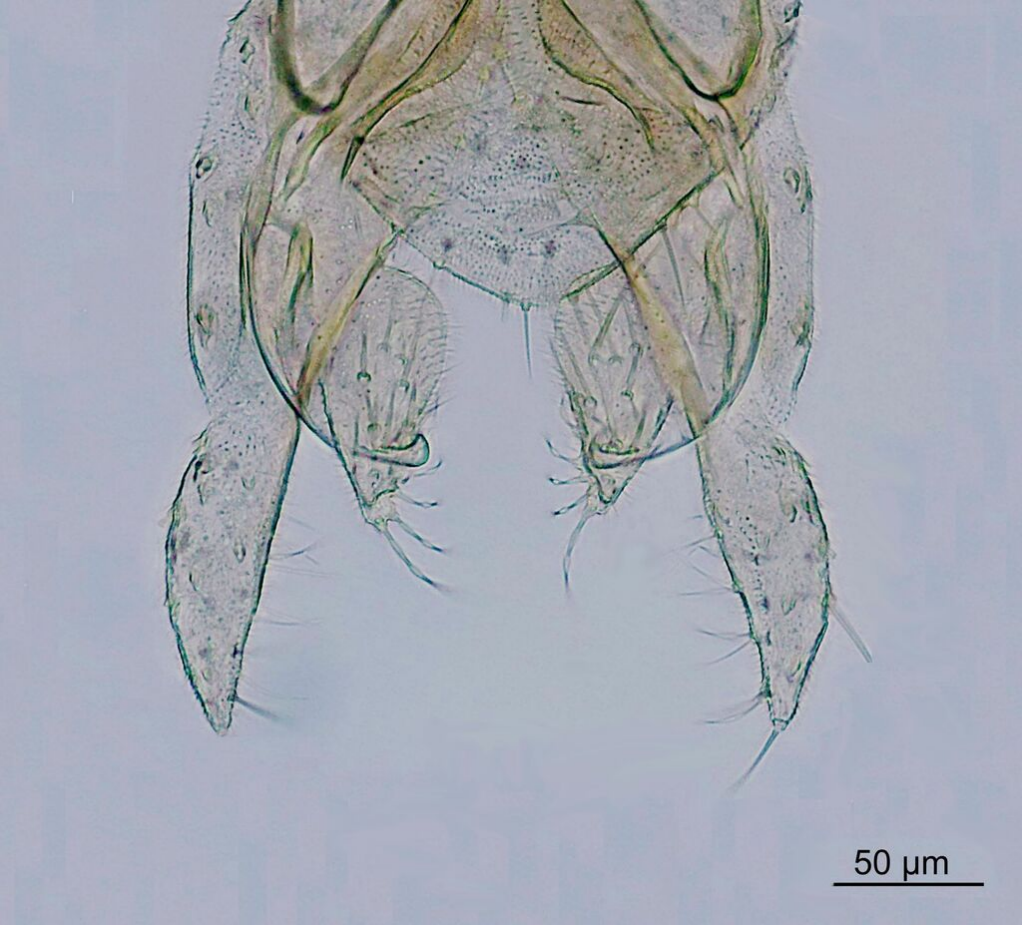
*Manoapahayokeensis* Jacobsen & Perry, male. Hypopygium, dorsal.

This species was originally described from the Everglades in Florida, USA and has previously been recorded from Monte Blanco, in the eastern parts of the Dominican Republic (Silva et al. 2015, Jacobsen and Perry 2002). During the present project, two males (Fig. 3) and two females were collected with sweep nets at Rio Blanco, in the central part of the Dominican Republic. The specimens were collected next to a small, approximately 6 m wide, fast flowing river Rio Blanco.

#### Notes

COI sequences of *M.pahayokeensis* were deposited in GenBаnk under accession numbers OR670329 and OR670330 (obtained from specimens "a" and "b", respectively).

## Discussion

The description of a new species of *Pseudochironomus* together with additional records of *Manoapahayokeensis* indicate a potentially higher unrecorded diversity of Pseudochironomini in Hispaniola and the Greater Antilleans.

## Supplementary Material

XML Treatment for Pseudochironomus ruthae

XML Treatment for Manoa pahayokeensis

## Figures and Tables

**Table 1.** Lengths (in µm) and proportions of leg segments in *Pseudochironomusruthae* Andersen & Baranov **sp. nov.**, male (n = 1).

	fe	ti	ta_1_	ta_2_	ta_3_	ta_4_	ta_5_	LR	BV	SV	BR
p_1_	980	1193	1095	507	409	286	131	0.918	2.454	1.985	2.18
p_2_	1087	1062	547	302	221	163	98	0.515	3.438	3.925	2.04
p_3_	1144	1201	703	400	335	204	114	0.585	2.891	3.337	2.12
